# Reflections on the 2nd International Congress on NanoBioEngineering 2020

**DOI:** 10.3389/fbioe.2021.648634

**Published:** 2021-02-01

**Authors:** José Rubén Morones-Ramírez

**Affiliations:** ^1^Facultad de Ciencias Químicas, Universidad Autónoma de Nuevo León, UANL, San Nicolás de los Garza, Mexico; ^2^Centro de Investigación en Biotecnología y Nanotecnología, Universidad Autónoma de Nuevo León, Parque de Investigación e Innovación Tecnológica, Apodaca, Mexico

**Keywords:** CINBI 2020, biotechnology, nanotechnology, nanobioengineering, synthetic biology, scientific communication

## Introduction

Nanotechnology and biotechnology are two of the fields that have generated the most “hype” within the last 20 years regarding their potential contribution to technological developments. Nanotechnology pursues the capability to design and synthesize nanometric structures and machines in a lego-like fashion, where each building block is an individual molecule. Biotechnology has the ultimate goal of designing, constructing, and using living organisms, or parts of them, for different applications. Therefore, it is fascinating and worth exploring where these fields can benefit from one another, and the field of NanoBioEngineering emerges at the interface of these fields. Nanobioengineering sees the great potential for biotechnology to take advantage of the powerful characterization tools and sophisticated synthesis developed within the field of nanotechnology to construct nanostructures and devices that interface organic and inorganic systems. On the other hand, biotechnology offers nanobioengineering the understanding of the genetic circuitry and biochemical processes that allow life in living organisms. Additionally, the solution to achieving organization of synthesized nanobiostructures lies in studying how biological systems have mastered self-assembly to build hierarchical systems through weak intramolecular forces.

The beforementioned applications and technological advances of NanoBioEngineering were exhibited at the 2nd International Congress on NanobioEngineering (CINBI2020), which took place on the virtual premises of the Centro de Investigacion en Biotecnologia y Nanotecnología (CIByN) at the Facultad de Ciencias Químicas in the Universidad Autónoma de Nuevo León in Monterrey, Mexico (24th−30th October, 2020). CINBI2020 brought together, virtually, scientists from different fields working on NanoBioEngineering and highlighted the current state of the art of seven thematic areas. More importantly, Science communication was a thematic area we decided to include, due to the times we are currently facing, where the importance of disseminating scientific thinking and scientifically validated information is critical. In this opinion, I highlight the Keynote Conferences and some of the works that were found of high relevance and which I believe have an impact on the advances of NanoBioEngineering toward the development of knowledge and technology that contribute toward the advancement of society.

## Discussion

Pardee et al. gave the inaugural keynote of the CINBI2020 and initiated the thematic area of bioprocess and synthetic biology. Pardee et al. coined the concept of living nanotechnology, describing how nano and bioengineering will converge in many ways but emphasized the development of novel low-cost detection systems for complex diseases, including COVID-19, Zika, and Chikungunya. Incredible technologies are being developed in his research group, including paper-based diagnostics and wearables, such as masks that incorporate novel CRISPR/CAS9-based modules that allow them to detect and discriminate viral strains with a single-base resolution. Therefore, these technologies can detect infectious agents such as COVID-19 in a persons' breath (Pardee et al., 2016). Next, Carbonell et al. discussed the potential of synthetic biology and the Design-Build-Test-Learn strategy to augment the pipeline for discovery and optimization of biological pathways that disembark in constructing microbial biofactories with highly optimized parameters to produce the next generation of novel antibiotics and fine chemicals (Carbonell et al., 2018). Afterwards, Duros et al. discussed in his keynote the advances made in his laboratory toward the design of liquid handling robots that obey machine learning algorithms that probe and predict conditions at which complex and large molecules can self-assemble and grow. Such advances have a wide array of applications since they allow the characterization of chemical systems and open a window to optimize industrial processes (Duros et al., 2017). Then, Laohakunakorn presented advances in developing computer-aided microfluidics that harbor cell-free gene expression systems that can be harnessed to produce different chemicals *in vitro* (Laohakunakorn, 2020). To complement the thematic area, speakers and presenters showed exciting scientific advances in gene essentiality, which will benefit the development of more efficient biological trajectories. Also, they exposed their technologies in the repurposing of agroindustrial waste, design, and optimization of complex genetic circuitry and synthetic ecologies to produce compounds for environmental, energy, and medical applications.

The thematic area on nanotechnology was inaugurated by Beliveau et al. where he described the state of the art of single-molecule detection systems, based on DNA nanotechnology, and how they can be used to detect and identify single molecules to monitor gene expression in biological processes on a cell by cell basis (Beliveau et al., 2015). Next, Sosnik et al. described different technologies developed toward nanostructures' active targeting in biomedical applications. One particular application exhibited in-depth was their advances in nanodrug delivery systems capable of encapsulating and releasing antituberculosis drugs to achieve a more effective, compliant, and affordable pharmacotherapy against this infectious disease (Sosnik et al., 2010). Additional works presented in the oral and poster presentations included studies on cytotoxicity and genotoxicity of nanostructures; the development of nanosystems capable of monitoring and managing bacterial diseases in the agroindustry, and also details of novel nanosystems used in the efficient delivery of drugs and vaccines were discussed. In addition other works presented involved synthesis of nanosystems capable of catalyzing chemical and biochemical reactions that led to either the assembly of proteins or virus-like particles' disaggregation. Finally, some of the works focused on developing bioremediation biosystems with the ability to remove heavy metals and transform them into high-value nanomaterials.

The thematic area of Nanomaterials started with keynotes imparted by Dr. Tom Ellis and Dr. Victor Manuel Castaño Meneses. Gilbert and Ellis emphasized the broad applications of synthetic biology in developing complex dynamic materials and how, through DNA, they can be programmed different functions (Gilbert and Ellis, 2018). Moreover, both Dr. Castaño and Dr. Ellis stressed the importance of exploring natural biological materials since they exhibit incredible physical and chemical properties that allow them to sense and respond to the environment, self-assemble, control their morphologies, and self-repair. Fernández-Cervantes et al. mentioned how in his laboratory, they could mimic the human skin motivated by the jellyfish's similar composition and architecture (Fernández-Cervantes et al., 2020). Both keynotes had the underlying message that natural materials must be sources of inspiration for scientists and nanobioengineering to design, develop, and synthesize our future materials. In the oral and poster presentations, the speakers talked about the strategies to develop and produce, through low-cost and environmentally friendly synthesis, nanostructured materials with applications in treating different diseases, including those with applications as antimicrobials, one of the main health issues society will confront soon. In addition, novel nano and nanobiomaterials with a wide array of sizes and morphologies were presented for their use as nanosensors to detect heavy metals and pollutants, contrasts in imaging technologies, as opto-thermal energy converters, and as biocatalysts for H_2_ production for the energy sector.

Computational Analysis and Mathematical Models was a thematic area that was addressed by Fernandes et al. in his keynote focused on the evolution of the fields of metabolomics and data mining that led toward the development of pathways and more complex biological instructions, which can now be used to elucidate aberrant pathways and metabolic dysfunction in disease (Fernandes et al., 2019). In the oral and poster sessions, the expert participants demonstrated theoretical models capable of predicting photocatalytic degradation of pollutants and simulating interactions of different compounds with the Sars-CoV2 spike protein. Works presented discussed *in-silico* design of edible vaccines against Sars-COV-2 and their ability to express them in tomato cells. Finally, incredible results were presented on the simulation of nanostructures' behavior under variable conditions and how external factors influence their physicochemical properties.

Four motivating keynotes complemented the thematic area on environment and bioeconomy with powerful messages. Conde-Pueyo et al. presented his keynote Bioremediation at a global scale from Petri dish to planet earth, where he discussed the different techniques and processes that have been developed at the interface of synthetic biology and nanotechnology toward the development of synthetic complex ecosystems that can be harnessed to perform diverse biological activities and if isolated, capable of creating their own closed environments and thrive under isolated synthetic conditions generated on earth; this has important applications to design and manipulate environments, such as those found in outer worlds, to create conditions hospitable to a specific lifeform (Conde-Pueyo et al., 2020). Next, François et al. discussed the challenges to cost-effective production from renewable carbon sources. He described different ways his research group has redesigned metabolic pathways in microorganisms to exploit them to convert non-edible renewable carbon sources into biofuels and commodity chemicals (François et al., 2019). Next, Malci et al. described how the combination of low-cost automation tools and complex molecular biology and genome editing methodologies would catapult our ability to strategically designed cell factories with a la carte biological pathways that produce specialty chemicals for all industrial sectors (Malci et al., 2020). Moreover, Velázquez-Hernández et al. discussed a specific example of using these technologies to revalorize glycerol, a byproduct of biodiesel production, to transform it and use it as fuel (Velázquez-Hernández et al., 2020). This section was completed with the experts who exposed their works in oral and poster presentations, touching topics from the production of wild-type crops to mitigate climate change to the development of novel biocatalyst bioreactors designed to repurpose agroindustrial waste and convert it to bioenergy. The exposed research also reported on novel autochthonous extremophile microorganisms and plant-microbe interactions that led to the development of systems for bioremediation of pollutants and pathogen control in crop production.

Three keynote speakers opened the thematic area focused on pharmacology and toxicology. Lopez et al. described different nano-based therapeutics that controlled drug delivery in *in vivo* patients suffering from brain glioma. Her work designed catalytic nanotherapies that suppressed tumors with more efficiency than commercially used cancer treatments. Her talk was inspiring and emphasized the vast avenue of new nano-pharmaceuticals being developed to treat other cancer types (Lopez et al., 2011). Next, León-Buitimea et al. talked about the role of nanobioengineering in the development of smart therapeutics with programmed dynamic responses to target, release, and clear therapeutics in an efficient manner in the treatment of infectious diseases caused by virus and bacteria (León-Buitimea et al., 2020). Finally, Correa et al. described the advances in her laboratory toward the design of hybrid composite nanoparticles that integrate enzymes to incorporate catalytic and superparamagnetic behaviors that would aid in designing drug delivery systems (Correa et al., 2019). The session was complemented with participants who exposed their research work on developing new drug delivery systems based on nanobioengineering to tackle different diseases, including tuberculosis, viral infections, including COVID-19, and cancer. Moreover, several participants highly stressed the need to expand the field of toxicity of nanomaterials into developing safe and useful nano-based therapeutics to treat different diseases.

The last thematic area presented at CINBI2020 was science communication, and it was started with a keynote by Davies et al. focused on the politics of public engagement and discussed how in order to have a positive impact and deliver the correct message in science communication, the strategies highly depend on the maturity and other aspects of society (Davies et al., 2019). Moreover, a powerful message was that science communication is entangled and shapes cultural stories and meanings, especially in an increasingly marked society by its reliance on science and technology. The scientists who participated in this thematic area exposed the mechanisms by which, through scientific photography, it is possible to transmit general messages to the population and create narrative tools for science communication and education. Moreover, the participants exhibited different settings and strategies in the forms of forums, webinars, and congress, which incentivize scientific information to the community in difficult times of society.

Overall, the speakers and participants shared and discussed their opinions, hypothesis, and original results. In particular, world experts in the field highlighted excellent topics regarding future technologies that will be developed in nanobioengineering and how they will impact the way humans live and interact. Moreover, the works presented at CINBI2020 give a lot of hope and satisfaction that the future technologies in NanoBioEngineering will increase the quality of life of our society but also, very importantly, those technologies will have less impact on the environment and therefore start the shift toward humans lives and activities being homeostatic with our planet. The seven thematic sessions presented at CINBI2020 demonstrate the vibrancy of a field that has considerably expanded in the last decade. Finally, CINBI2020 consolidated scientists with similar research interests and provided opportunities to conduct future collaborative studies and propose future fruitful and interdisciplinary research projects.

## Author Contributions

JM-R wrote and edited the entire opinion manuscript.

## Conflict of Interest

The author declares that the research was conducted in the absence of any commercial or financial relationships that could be construed as a potential conflict of interest.
